# The Association between Autoimmune Thyroid Disease and Ocular Surface Damage: A Retrospective Population-Based Cohort Study

**DOI:** 10.3390/jcm12093203

**Published:** 2023-04-29

**Authors:** Eric W. Lai, Ying-Hsuan Tai, Hsiang-Ling Wu, Ying-Xiu Dai, Tzeng-Ji Chen, Yih-Giun Cherng, Shih-Chung Lai

**Affiliations:** 1 University of Maryland School of Medicine, Baltimore, MD 21201, USA; 2 Department of Anesthesiology, Shuang Ho Hospital, Taipei Medical University, New Taipei City 23561, Taiwan; 3 Department of Anesthesiology, School of Medicine, College of Medicine, Taipei Medical University, Taipei 11031, Taiwan; 4 Department of Anesthesiology, Taipei Veterans General Hospital, Taipei 11217, Taiwan; 5 School of Medicine, National Yang Ming Chiao Tung University, Taipei 11221, Taiwan; 6 Department of Dermatology, Taipei Veterans General Hospital, Taipei 11217, Taiwan; 7 Department of Family Medicine, Taipei Veterans General Hospital, Taipei 11217, Taiwan; 8 Department of Family Medicine, Taipei Veterans General Hospital, Hsinchu Branch, Hsinchu 31064, Taiwan; 9 Department of Ophthalmology, Shuang Ho Hospital, Taipei Medical University, New Taipei City 23561, Taiwan; 10 Department of Ophthalmology, School of Medicine, College of Medicine, Taipei Medical University, Taipei 11031, Taiwan

**Keywords:** corneal ulcer, Graves’ ophthalmopathy, keratoconjunctivitis sicca, peripheral ulcerative keratitis, thyroid eye disease

## Abstract

Autoimmune thyroid diseases (ATDs) are potentially connected to lacrimal gland dysfunction and ocular surface disruption. This study aimed to evaluate the relationships between ATD, dry eye disease (DED), and corneal surface damage. In a matched nationwide cohort study, we used Taiwan’s National Health Insurance research database to compare the incidences of DED and corneal surface damage between subjects with and without ATD. Multivariable Cox proportional hazards regression models were used to estimate the adjusted hazard ratio (aHR) and 95% confidence interval (CI) for the ophthalmological outcomes. A total of 50,251 matched pairs with 748,961 person-years of follow-up were included for analysis. The incidence of DED was 16.37 and 8.36 per 1000 person-years in the ATD and non-ATD groups, respectively. ATDs were significantly associated with increased DED (aHR: 1.81, 95% CI: 1.73–1.89, *p* < 0.0001). This association was generally consistent across the subgroups of age, sex, different comorbidity levels, and use of systemic corticosteroids or not. Furthermore, patients with ATD had a higher risk of corneal surface damage compared with non-ATD subjects (aHR: 1.31, 95% CI: 1.19–1.44, *p* < 0.0001), including recurrent corneal erosions (aHR: 2.00, 95% CI: 1.66–2.41, *p* < 0.0001) and corneal scars (aHR: 1.26, 95% CI: 1.01–1.59, *p* = 0.0432). Other independent factors for corneal surface damage were age, sex, diabetes mellitus, Charlson Comorbidity Index scores, and use of systemic corticosteroids. Our results suggested that ATDs were associated with higher risks of DED and corneal surface damage. Considering the high prevalence of ATD, prophylactic and therapeutic strategies should be further developed to prevent irreversible vision loss in this susceptible population.

## 1. Introduction

Dry eye disease (DED) is a multifaceted disorder of the corneal surface with the pathogenesis of decreased tear production, excessive evaporation of tears, and ocular surface inflammation [1]. DED is a leading cause of ophthalmology clinic visits, and a meta-analysis recently reported an estimated prevalence of 8.1% amongst adults in the United States [2]. The prevalence of DED increases with age and is higher in females than males [2,3]. In severe cases, DED can progress into corneal ulcers and perforations [4,5]. Hung et al. recently demonstrated that patients with aqueous-deficient DED had an increased risk of corneal surface damage in a long-term follow-up of a population-based cohort [6]. Mounting evidence has shown that systemic autoimmune diseases are closely connected to DED and corneal inflammation, such as rheumatoid arthritis and systemic lupus erythematosus [7].

Autoimmune thyroid diseases (ATDs) are the most common organ-specific autoimmune disorders worldwide, affecting approximately 2 to 5% of the global population, with females in the majority [8,9]. Graves’ disease and Hashimoto thyroiditis are the most frequent diagnoses among ATDs [8,9]. The etiology of ATD is complex and multifactorial, and a combination of genetic predisposition and environmental factors are thought to be possible causes [8,9]. The loss of immunological tolerance and consequent autoimmune attacks to the thyroid tissue are responsible for the follicular destruction and thyroid function abnormality [10]. The clinical and pathological features of ATD are various depending on whether a state of hyperthyroidism, hypothyroidism, or both predominate in patients [11]. In addition to thyroid tissue damage and functional changes, ATD may also have a deleterious impact on other systems, such as the eye [7]. In a retrospective observation, Gupta et al. reported that 19% of patients with DED symptoms had occult thyroid eye diseases or other rheumatological disorders, suggesting a strong association between DED and ATD [12]. Lacrimal gland destruction, tear film instability, and eyelid abnormality are also common ocular pathologies among patients with ATDs, sharing similar pathophysiology with DED [13,14,15,16,17,18,19,20]. In addition to anatomical changes (e.g., proptosis and corneal exposure), corneal surface inflammation potentially contributes to these ocular symptoms in ATD [20,21].

However, few studies have described the incidence and epidemiology of DED associated with ATD using population-based datasets. Most previous studies focused on the relationship between ATD and primary Sjögren’s syndrome (SS), and the risk of DED in patients with ATD remains unclear [22,23,24,25]. In addition, since the incidence of corneal surface damage is relatively low, the small patient samples in previous studies (<200 ATD subjects) precluded the analyses of corneal injury risk associated with ATD [22,23,24,25]. Related risk factors are also largely unknown in the literature. Therefore, we conducted a retrospective, nationwide, population-based, matched cohort study to investigate the putative associations between ATD, DED, and corneal surface damage. There were two objectives in this study. First, we aimed to clarify whether the diagnosis of ATD is associated with a long-term risk of DED or corneal surface damage using a large population-based cohort. Second, we sought to identify associated risk factors for serious corneal diseases among patients with ATD. Based on current evidence [13,14,15,16,17,18,19,20,21], we hypothesized that ATDs were associated with both increased DED and corneal surface damage in this population-based cohort.

## 2. Material and Methods

### 2.1. Data Source

This study was approved by the Taipei Medical University-Joint Institutional Review Board (approval no. TMU-JIRB-N202210011; date of approval: 6 October 2022). Written informed consent was waived by the Institutional Review Board because the research data were based on deidentified information. All methods were performed in accordance with the Declaration of Helsinki 2013 and related research guidelines [26]. In the present study, we utilized the data from the National Health Insurance (NHI) research database in Taiwan. Taiwan’s National Health Insurance program was implemented in March 1995 and covers more than 99% of 23.3 million Taiwanese residents and foreigners working or studying in Taiwan. A detailed description of this database was given in our previous articles [27,28,29]. In this study, we enrolled subjects from the 3 Longitudinal Health Insurance Databases (LHID2000, LHID2005, and LHID2010), which contain original claims data of 1 million randomly sampled beneficiaries from the original NHI research database in the years 2000, 2005, and 2010, respectively [30].

### 2.2. Study Population and Definition

We consecutively included patients who had at least 2 endocrinology or rheumatology clinic visits with diagnoses of ATD from 1 January 2002 to 30 June 2013. The International Classification of Diseases, 9th Revision, Clinical Modification (ICD-9-CM) codes were employed to determine the diagnoses of ATD, comorbidities, and ocular diseases (Supplementary Table S1). The index date was defined as the date of the first diagnosis of ATD. Exclusion criteria were any previous diagnoses of DED, corneal ulcers, recurrent corneal erosion, corneal scars, interstitial and deep keratitis, corneal neovascularization, ocular burns, or open globe injury by certified ophthalmologists before the index date. Subjects were also excluded if they had any prescriptions of eye lubricants before the index date or died during the follow-up period.

### 2.3. Ocular Surface Disease

The primary outcome was DED, defined as the diagnosis (ICD-9-CM codes 370.33, 372.53, 375.15, and 710.2) made at least twice, along with the prescriptions of cyclosporine ophthalmic emulsion by certified ophthalmologists. According to the NHI regulations, cyclosporine ophthalmic emulsion treatment was reimbursable when Schirmer test scores were lower than 5 mm in 5 min [6]. The secondary outcomes were secondary SS and severe forms of corneal surface damage, which included any diagnosis of corneal ulcers, recurrent corneal erosion, or corneal scars made twice in the ophthalmology care service. The NHI research database did not contain detailed data on clinical medical or surgical treatments for corneal surface diseases, which precluded qualitative or quantitative assessments of DED.

### 2.4. Covariates for Adjustment

Coexisting diseases were collected according to the ICD-9-CM codes of physicians’ diagnoses within 24 months before the index date, including hypertension, diabetes mellitus, coronary artery disease, chronic obstruction pulmonary disease, chronic liver disease, chronic kidney disease, cerebrovascular disease, major depressive disorder, anxiety disorder, sleeping disorder, and cancer (Supplementary Table S1) [31]. The Charlson Comorbidity Index was employed to categorize the comorbidities of included patients [32]. The concurrent prescription of systemic corticosteroids within 6 months after the index date was also examined to reflect the disease severity of ATD. We calculated the total numbers of hospital admissions and emergency room visits within 24 months before the index date to represent the level of healthcare resource utilization among the included subjects.

### 2.5. Statistical Analysis

Each ATD subject was matched to a non-ATD control using the propensity score methodology. Briefly, a non-parsimonious multivariable logistic regression model was implemented to obtain a propensity score for ATD and non-ATD subjects. A nearest neighbor matching algorithm with a caliper width of 0.2 standard deviation of the log odds of the estimated propensity score and without replacement was used to balance the distributions of age, sex, and monthly insurance premium between the 2 groups [33]. An absolute standardized mean difference (ASMD) was used to compare the baseline patient characteristics between groups [34]. Imbalance was defined as an ASMD value higher than 0.1. Multivariable Cox proportional hazards regression models were used to calculate the adjusted hazard ratio (aHR) and 95% confidence interval (CI) for the ophthalmological outcomes. The factors controlled in the multivariable models were age, sex, monthly insurance premium, comorbidities, Charlson Comorbidity Index scores, use of systemic corticosteroids, number of hospitalizations, and number of emergency room visits. The Kaplan–Meier methods and log-rank tests were used to demonstrate the cumulative incidence difference in the ophthalmological outcomes between the two groups. We also conducted subgroup analyses by age ≥ or <65 years, male or female, varying Charlson Comorbidity Index scores, and use of systemic corticosteroids or not. A two-sided *p*-value of <0.05 was considered statistically significant. All the statistical analyses were conducted using Statistics Analysis System (SAS), Version 9.4 (SAS Institute Inc., Cary, NC, USA).

## 3. Results

### 3.1. Baseline Patient Characteristics

A total of 50,251 matched pairs with 748,961 person-years of follow-up were included for analysis (Supplementary Figure S1). The median follow-up time was 7.8 years (interquartile range: 4.3–10.6, range: 0.1–11.9) in ATD subjects and 8.3 years (interquartile range: 4.8–10.8, range: 0.1–11.9) in non-ATD controls. Table 1 shows the distributions of baseline patient characteristics in the ATD and non-ATD groups. Notably, patients with ATD were more likely to have more comorbidities, use systemic corticosteroids, and have greater numbers of hospital admissions and emergency room visits.

### 3.2. Dry Eye Disease

**Figure 1:**
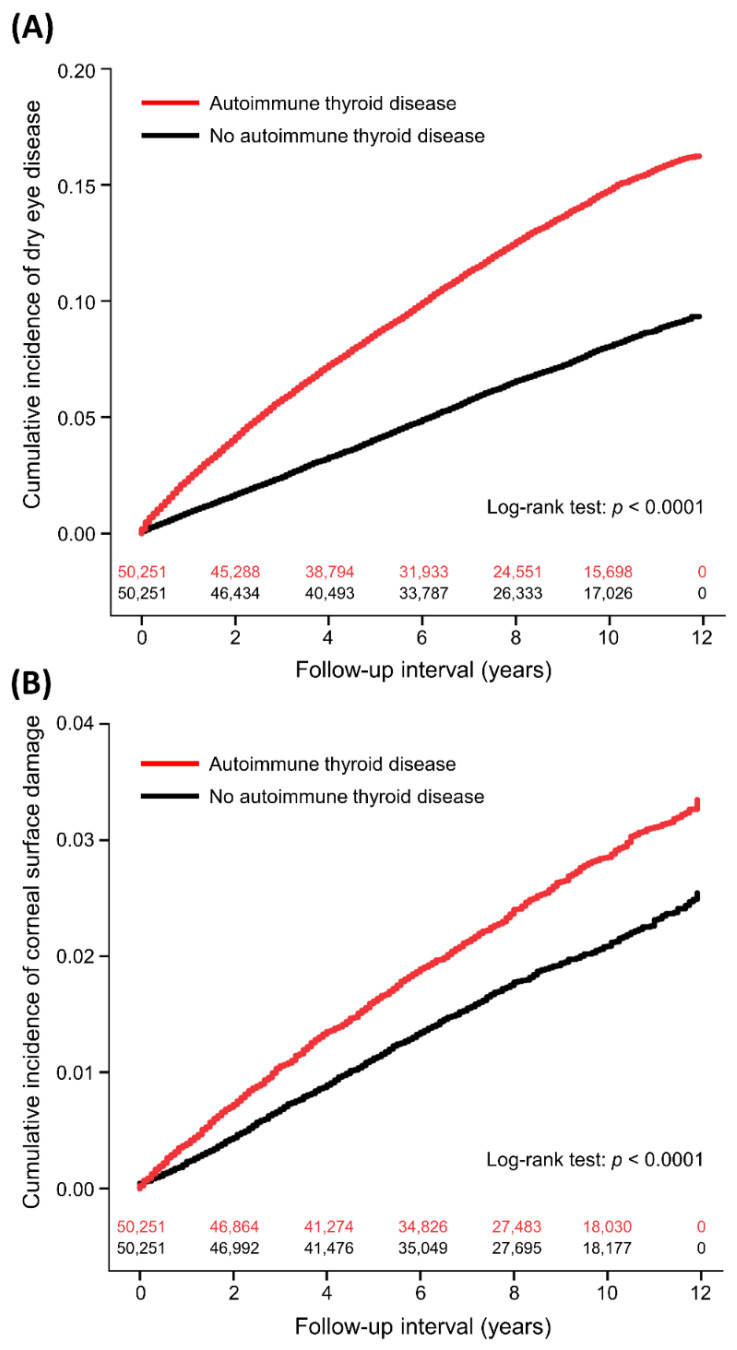
Cumulative incidence of dry eye disease (**A**) and corneal surface damage (**B**) in patients with and without autoimmune thyroid diseases with number of subjects at risk.

The period incidence of DED was 16.37 and 8.36 per 1000 person-years in the ATD and non-ATD groups, respectively (Table 2). The interval between enrollment and DED diagnoses was median 3.3 (interquartile range: 1.4–6.2) years in the ATD group and 4.3 (2.0–6.9) years in the non-ATD group (*p* < 0.0001). Table 3 shows the aHR and 95% CI for DED in the univariate and multivariable models. In the multivariable model, ATDs were significantly associated with increased DED (aHR: 1.81, 95% CI: 1.73–1.89, *p* < 0.0001). Figure 1A shows the cumulative incidence of DED in the two groups. ATDs were also associated with a higher incidence of secondary SS (aHR: 2.24, 95% CI: 2.01–2.50, *p* < 0.0001). Other independent factors for DED were age (aHR: 1.03), sex (male vs. female, aHR: 0.52), monthly insurance premium (USD 501–800 vs. 0–500, aHR: 0.86; USD ≥801 vs. 0–500, aHR: 1.16), hypertension (aHR: 0.93), coronary artery disease (aHR: 1.12), chronic liver disease (aHR: 1.23), major depressive disorder (aHR: 1.26), anxiety disorder (aHR: 1.38), sleeping disorder (aHR: 1.19), use of systemic corticosteroids (aHR: 1.32), and number of hospitalizations (1 vs. 0, aHR: 0.90; 2 vs. 0, aHR: 0.97; ≥3 vs. 0, aHR: 0.68). Subgroup analyses showed consistent associations between ATD and DED across the subgroups of age ≥ or <65 years, male or female, different Charlson Comorbidity Index scores, and use of systemic corticosteroids or not (Table 4).

### 3.3. Corneal Surface Damage

The incidence of corneal surface damage was 2.98 and 2.17 per 1000 person-years in the ATD and non-ATD subjects, respectively (Table 2). The time to corneal surface damage was median 3.7 years (interquartile range: 1.6–6.5) in the ATD group and 4.2 years (interquartile range: 2.0–6.8) in the non-ATD group (*p* = 0.0196). After adjusting for covariates, ATD was independently associated with corneal surface damage (aHR: 1.31, 95% CI: 1.19–1.44, *p* < 0.0001; Table 5 and Figure 1B), including recurrent corneal erosion (aHR: 2.00, 95% CI: 1.66–2.41, *p* < 0.0001) and corneal scar (aHR: 1.26, 95% CI: 1.01–1.59, *p* = 0.0432). Other independent factors for corneal surface damage were age (aHR: 0.996), sex (male vs. female, aHR: 0.87), diabetes mellitus (aHR: 1.31), Charlson Comorbidity Index scores (1 vs. 0, aHR: 1.27; 2 vs. 0, aHR: 1.14; ≥3 vs. 0, aHR: 0.12), and use of systemic corticosteroids (aHR: 1.26).

## 4. Discussion

In this 12-year population-based cohort study, our findings suggested that ATDs were significantly associated with increased risks of DED and corneal surface injury. This association was generally consistent across the various subgroups of age, sex, use of systemic corticosteroids, and varying comorbidity levels and indices. Our results highlight the importance of routine ophthalmology examinations and close follow-up visits to prevent potential corneal complications among patients with ATD.

There are few studies in the literature that have compared the incidence of DED and corneal surface damage between ATD and non-ATD patients. In a cohort study based in the Netherlands, DED was associated with comorbidities in almost every body system, particularly in young adults [35]. Another study proposed that thyroid diseases have the potential to be a risk factor for DED [36]. In a recent meta-analysis, DED was associated with older age, female sex, cataract surgery, contact lens wear, psychiatric illnesses, sleep apnea, hypertension, diabetes mellitus, cardiovascular disease, and more [3]. In another multicenter cohort study, severe DED signs were associated significantly with SS, facial rosacea, rheumatoid arthritis, peripheral arterial disease, and daily smoking history. Other systemic conditions, such as thyroid dysfunction, were not found to be associated significantly with DED signs [37]. On the contrary, our results suggested that patients with thyroid diseases, particularly those due to autoimmune causes, had an increased risk of DED and corneal diseases. Additionally, our study identified several risk factors for DED and corneal diseases associated with ATD which have not been reported in previous studies [3,36,37,38]. These findings may be helpful in strategic risk stratification to address early diagnosis and prevention of severe ocular complications.

The pathogenesis of DED and corneal disease in ATD is still not well understood. Traditionally, DED as part of ocular surface diseases in the setting of ATD is thought to be due to anatomical changes from proptosis and corneal exposure [20]. Growing evidence, however, suggests that this is a multifactorial phenomenon, with ocular surface inflammation playing a key role in DED development seen in ATD patients [20,21]. In thyroid eye disease, some studies have proposed that orbital inflammation early in the disease manifests as dry eye symptoms, while increased exposure serves as the catalyst in more severe signs of dry eye symptoms later in the disease course [39]. Additionally, studies have demonstrated that inflammatory conjunctival cytokines play a role in the pathogenesis of ATD-associated DED. These include interleukin (IL)-1a, IL-1b, and IL-6 [40].

Furthermore, it is well documented in the literature that patients with thyroid eye disease have been reported to have more severe meibomian gland dysfunction due to loss of Meibomian gland structure in the eyelids [41,42]. This phenomenon of decreased basal tear secretion, impaired meibum expression, and incomplete blinking in these patients may offer potential explanations for the associations between ATD, DED, and corneal surface injury [16].

These studies suggest that there may be a temporal component to the mechanism and severity of DED and corneal surface damage in connection with ATD. Therefore, timely referral to an ophthalmologist for a comprehensive eye examination is an important first step in management. Early recognition and diagnosis are vital in preventing progressive dry eye symptoms and corneal injury causing irreversible vision loss. Our findings strongly extend support for the associations between ATD, DED, and corneal surface disease, warranting further studies to clarify the biological mechanism and to evaluate effective prophylactic and therapeutic management for these patients.

The present study had several strengths to delineate the association between ATD and DED or corneal diseases. Our large-scale population-based cohort study increased statistical power and our data provided reliable epidemiological evidence and generalizability. The included subjects were followed up for a maximum of 12 years to evaluate the temporal associations between ATD, DED, and corneal surface diseases. Our analyses also identified several risk factors previously unreported to support the association between ATD and DED.

Our study also had unique limitations. First, since the NHI research database was restricted to diagnosis- and treatment-based data, environmental factors, lifestyle habits (contact lens wearing and cigarette smoking), biochemical profiles (autoimmune and inflammatory markers), detailed clinical data (pharmacological or surgical treatments), and objective physical measurements (ophthalmic test results) were not available and could not be adjusted for in the statistical model. The level of ATD severity and/or activity in our subjects could not be further clarified or evaluated. We did not analyze previous ophthalmic surgeries for cataract or glaucoma in the included subjects, which potentially affected the development of ocular surface diseases. However, the proportion of these patients should be low due to the relatively young population in this study (39.8 years old on average). Second, the NHI research database did not contain parameters of proptosis or other anatomical abnormalities. Therefore, it was unclear whether the DED and corneal injury associated with ATD were due to anatomical or biochemical etiologies. Third, patients’ thyroid functions could not be analyzed due to data unavailability. Fourth, we used the prescriptions of ophthalmic cyclosporine to define the diagnosis of DED, which might underestimate the incidence of DED. Fifth, the ascertainment of DED in our analyses did not include patients who were prescribed ophthalmic cyclosporine by self-payment. Lastly, our cohort was followed up until the end of 2013, due to regulations of the NHI research database.

## 5. Conclusions

This 12-year population-based cohort study revealed that patients with ATD had an increased risk of DED and corneal surface damage compared with non-ATD controls. We identified several risk factors for DED and corneal surface injury, suggesting the importance of early identification and prevention of serious corneal complications in patients with ATD. Routine ophthalmology evaluation and close follow-up are needed to prevent vision-threatening sequelae in this susceptible population. Further studies are warranted to evaluate optimal therapeutic management in patients with ATD.

## Figures and Tables

**Table 1:** Baseline characteristics of subjects with and without autoimmune thyroid diseases.

	ATD *n* = 50,251		Non-ATD *n* = 50,251		ASMD
Age (years), mean (SD)	39.8	15.0	39.8	15.0	<0.0001
Sex, male, *n* (%)	11,065	22.0	11,065	22.0	<0.0001
Insurance premium (USD/month), *n* (%)					<0.0001
0–500	17,022	33.9	17,022	33.9	
501–800	16,212	32.3	16,212	32.3	
≥801	17,017	33.9	17,017	33.9	
Coexisting diseases, *n* (%)					
Hypertension	6661	13.3	4314	8.6	0.2684
Diabetes mellitus	3500	7.0	2034	4.1	0.3163
Coronary artery disease	3046	6.1	1387	2.8	0.4528
Chronic obstructive pulmonary disease	2268	4.5	1259	2.5	0.3360
Chronic liver disease	4577	9.1	2415	4.8	0.3780
Chronic kidney disease	327	0.7	177	0.4	0.3401
Cerebrovascular disease	1248	2.5	731	1.5	0.3007
Major depressive disorder	704	1.4	304	0.6	0.4674
Anxiety disorder	7681	15.3	2992	6.0	0.5774
Sleeping disorder	6231	12.4	3227	6.4	0.3992
Cancer	1314	2.6	819	1.6	0.2662
Charlson Comorbidity Index					0.0642
0	45,572	90.7	46,587	92.7	
1	3300	6.6	2594	5.2	
2	1179	2.4	854	1.7	
≥3	200	0.4	216	0.4	
Use of systemic corticosteroids, *n* (%)	8935	17.8	6222	12.4	0.2346
Number of hospitalizations, *n* (%)					0.1023
0	44,033	87.6	45,770	91.1	
1	4726	9.4	3443	6.9	
2	997	2.0	677	1.4	
≥3	495	1.0	361	0.7	
Number of emergency room visits, *n* (%)					0.2209
0	37,162	74.0	41,577	82.7	
1	8251	16.4	6066	12.1	
2	2684	5.3	1564	3.1	
≥3	2154	4.3	1044	2.1	

Abbreviations: ASMD = absolute standardized mean difference; ATD = autoimmune thyroid disease; SD = standard deviation; USD = United States Dollar.

**Table 2:** Incidences of dry eye disease, secondary Sjögren’s syndrome, and corneal surface damage for patients with and without autoimmune thyroid diseases.

	ATD *n* = 50,251		Non-ATD *n* = 50,251		Outcome Risk		
Study Outcome	Incident Case	Incidence per 1000 Person-Years	Incident Case	Incidence per 1000 Person-Years	IRR	aHR (95% CI) ^†^	*p*
Dry eye disease	5996	16.37	3198	8.36	1.96	1.81 (1.73–1.89)	<0.0001
Sjögren’s syndrome-associated	1292	3.30	519	1.31	2.52	2.24 (2.01–2.50)	<0.0001
Non-Sjögren’s syndrome-associated	4704	12.84	2679	7.00	1.83	1.72 (1.63–1.80)	<0.0001
Sjögren’s syndrome	1292	3.30	519	1.31	2.52	2.24 (2.01–2.50)	<0.0001
Corneal surface damage	1168	2.98	854	2.17	1.37	1.31 (1.19–1.44)	<0.0001
Corneal ulcer	611	1.56	520	1.32	1.18	1.10 (0.97–1.24)	0.1565
Recurrent corneal erosion	377	0.96	183	0.46	2.09	2.00 (1.66–2.41)	<0.0001
Corneal scar	186	0.47	154	0.39	1.21	1.26 (1.01–1.59)	0.0432

Abbreviations: aHR = adjusted hazard ratio; ATD = autoimmune thyroid disease; CI = confidence interval; IRR = incidence rate ratio. ^†^ Adjusted for age (continuous), sex, monthly insurance premium (categorical), coexisting diseases, Charlson Comorbidity Index score, use of systemic corticosteroids, and numbers of hospitalizations and emergency room visits.

**Table 3:** Univariate and multivariable analyses for dry eye disease.

	Univariate			Multivariable		
	cHR	95% CI	*p*	aHR	95% CI	*p*
Autoimmune thyroid disease	1.95	1.87–2.04	<0.0001	1.81	1.73–1.89	<0.0001
Age (years)	1.03	1.03–1.03	<0.0001	1.03	1.03–1.03	<0.0001
Sex, male vs. female	0.56	0.53–0.60	<0.0001	0.52	0.49–0.56	
Insurance premium (USD/month)			<0.0001			<0.0001
501–800 vs. 0–500	0.71	0.67–0.74	<0.0001	0.86	0.82–0.91	<0.0001
≥801 vs. 0–500	0.78	0.75–0.82	<0.0001	1.16	1.10–1.22	<0.0001
Coexisting diseases						
Hypertension	1.85	1.75–1.96	<0.0001	0.93	0.87–1.00	0.0388
Diabetes mellitus	1.79	1.67–1.93	<0.0001	1.08	0.99–1.17	0.0801
Coronary artery disease	2.17	2.01–2.33	<0.0001	1.12	1.03–1.21	0.0106
COPD	1.77	1.62–1.94	<0.0001	1.09	1.00–1.20	0.0599
Chronic liver disease	1.69	1.58–1.81	<0.0001	1.23	1.15–1.32	<0.0001
Chronic kidney disease	1.67	1.30–2.15	<0.0001	0.98	0.76–1.26	0.8470
Cerebrovascular disease	1.84	1.63–2.07	<0.0001	0.93	0.82–1.06	0.2626
Major depressive disorder	1.95	1.66–2.28	<0.0001	1.26	1.07–1.48	0.0056
Anxiety disorder	2.10	1.99–2.21	<0.0001	1.38	1.30–1.47	<0.0001
Sleeping disorder	1.91	1.80–2.03	<0.0001	1.19	1.12–1.27	<0.0001
Cancer	1.68	1.49–1.89	<0.0001	1.09	0.96–1.23	0.1923
Charlson Comorbidity Index			<0.0001			0.5526
1 vs. 0	1.50	1.39–1.61	<0.0001	0.95	0.88–1.03	0.1957
2 vs. 0	1.45	1.29–1.64	<0.0001	1.00	0.88–1.13	0.9583
≥3 vs. 0	1.24	0.93–1.64	0.1405	0.90	0.68–1.20	0.4874
Use of systemic corticosteroids	1.47	1.40–1.55	<0.0001	1.32	1.25–1.39	<0.0001
Number of hospitalizations			0.0003			0.0014
1 vs. 0	1.06	0.98–1.15	0.1305	0.90	0.83–0.97	0.0068
2 vs. 0	1.36	1.17–1.58	<0.0001	0.97	0.83–1.13	0.7025
≥3 vs. 0	1.14	0.90–1.43	0.2780	0.68	0.53–0.87	0.0020
Number of emergency room visits			<0.0001			0.4599
1 vs. 0	1.09	1.02–1.15	0.0071	0.99	0.93–1.05	0.7328
2 vs. 0	1.20	1.08–1.32	0.0006	0.98	0.89–1.09	0.7527
≥3 vs. 0	1.20	1.06–1.35	0.0031	0.90	0.79–1.02	0.1094

Abbreviations: aHR = adjusted hazard ratio; COPD = chronic obstruction pulmonary disease; cHR = crude hazard ratio; USD = United States Dollar.

**Table 4:** Subgroup analyses of dry eye disease for patients with and without autoimmune thyroid diseases.

	ATD *n* = 50,251		Non-ATD *n* = 50,251		Outcome Risk		
Subgroup	Incident Case	Incidence per 1000 Person-Years	Incident Case	Incidence per 1000 Person-Years	IRR	aHR (95% CI) ^†^	*p*
All patients	5996	16.37	3198	8.36	1.96	1.81 (1.73–1.89)	<0.0001
Age ≥ 65 years	631	28.99	400	17.28	1.68	1.56 (1.36–1.78)	<0.0001
Age < 65 years	5365	15.57	2798	7.78	2.00	1.84 (1.75–1.93)	<0.0001
Male	829	10.50	398	4.89	2.15	1.95 (1.71–2.21)	<0.0001
Female	5167	17.98	2800	9.30	1.93	1.78 (1.70–1.87)	<0.0001
CCI score = 0	5242	15.86	2814	7.99	1.98	1.84 (1.75–1.93)	<0.0001
CCI score = 1	550	21.75	268	12.51	1.74	1.66 (1.42–1.93)	<0.0001
CCI score = 2	176	19.75	96	13.53	1.46	1.35 (1.03–1.75)	0.0278
CCI score ≥ 3	28	18.17	20	11.30	1.61	1.33 (0.69–2.56)	0.3954
Use of systemic corticosteroids	1365	21.20	533	11.06	1.92	1.78 (1.60–1.98)	<0.0001
No use of systemic corticosteroids	4631	15.34	2665	7.97	1.92	1.81 (1.72–1.91)	<0.0001

Abbreviations: aHR = adjusted hazard ratio; ATD = autoimmune thyroid disease; CCI = Charlson Comorbidity Index; CI = confidence interval; IRR = incidence rate ratio. ^†^: Adjusted for age (continuous), sex, monthly insurance premium (categorical), coexisting diseases, Charlson Comorbidity Index score, use of systemic corticosteroids, number of hospitalizations, and number of emergency room visits.

**Table 5:** Univariate and multivariable analyses for corneal surface damage.

	Univariate			Multivariable		
	cHR	95% CI	*p*	aHR	95% CI	*p*
Autoimmune thyroid disease	1.37	1.26–1.50	<0.0001	1.31	1.19–1.44	<0.0001
Age (years)	0.998	0.995–1.001	0.2272	0.996	0.993–1.000	0.0354
Sex, male vs. female	0.88	0.79–0.98	0.0218	0.87	0.78–0.98	0.0180
Insurance premium (USD/month)			0.6098			0.7713
501–800 vs. 0–500	1.05	0.94–1.17	0.3893	1.04	0.93–1.16	0.4958
≥801 vs. 0–500	1.00	0.90–1.11	1.0000	1.01	0.90–1.13	0.8773
Coexisting diseases						
Hypertension	1.02	0.88–1.18	0.7675	0.92	0.77–1.09	0.3355
Diabetes mellitus	1.37	1.15–1.63	0.0005	1.31	1.08–1.59	0.0065
Coronary artery disease	1.12	0.91–1.38	0.2931	1.02	0.81–1.28	0.8762
COPD	1.16	0.93–1.46	0.1888	1.08	0.85–1.36	0.5323
Chronic liver disease	1.12	0.95–1.33	0.1836	1.02	0.86–1.21	0.8312
Chronic kidney disease	0.72	0.32–1.60	0.4152	0.63	0.28–1.42	0.2637
Cerebrovascular disease	1.11	0.81–1.53	0.5038	1.03	0.74–1.44	0.8606
Major depressive disorder	1.47	1.00–2.15	0.0478	1.25	0.85–1.84	0.2643
Anxiety disorder	1.21	1.06–1.39	0.0063	1.05	0.90–1.22	0.5258
Sleeping disorder	1.23	1.06–1.43	0.0063	1.12	0.95–1.32	0.1738
Cancer	0.99	0.72–1.37	0.9616	0.97	0.70–1.34	0.8321
Charlson Comorbidity Index			0.0022			0.0056
1 vs. 0	1.28	1.09–1.51	0.0025	1.27	1.07–1.52	0.0065
2 vs. 0	1.13	0.85–1.50	0.4012	1.14	0.85–1.52	0.3750
≥3 vs. 0	0.11	0.02–0.79	0.0283	0.12	0.02–0.83	0.0318
Use of systemic corticosteroids	1.31	1.18–1.47	<0.0001	1.26	1.13–1.41	<0.0001
Number of hospitalizations			0.5917			0.1493
1 vs. 0	0.91	0.76–1.08	0.2757	0.82	0.68–0.98	0.0313
2 vs. 0	0.97	0.67–1.41	0.8700	0.82	0.56–1.21	0.3169
≥3 vs. 0	1.22	0.76–1.96	0.4187	0.97	0.59–1.60	0.8974
Number of emergency room visits			0.0224			0.2657
1 vs. 0	1.15	1.01–1.31	0.0301	1.11	0.98–1.27	0.1143
2 vs. 0	1.08	0.86–1.35	0.5367	1.01	0.80–1.28	0.9111
≥3 vs. 0	1.34	1.05–1.71	0.0186	1.21	0.93–1.57	0.1584

Abbreviations: aHR = adjusted hazard ratio; COPD = chronic obstruction pulmonary disease; cHR = crude hazard ratio; USD = United States Dollar.

## Data Availability

The data presented in this study are available on request from the corresponding author.

## Supplementary Materials

The following supporting information can be downloaded at: https://www.mdpi.com/article/10.3390/jcm12093203/s1, Table S1: ICD-9-CM codes of exposure factors, coexisting diseases, and study outcomes; Figure S1: Flow diagram for patient selection.
