# Supplementary material for: The Association between Autoimmune Thyroid Disease and Ocular Surface Damage: A Retrospective Population-Based Cohort Study
Source: J Clin Med. 2023 Apr 29;12(9):3203. doi: 10.3390/jcm12093203 (PMC10179488; doi:10.3390/jcm12093203)
Supplement: Supplementary file 1 [file jcm-12-03203-s001.zip › Supplementary Table S1.pdf]

Supplementary Table S1. ICD-9-CM codes of exposure factors, coexisting diseases, and study outcomes

**Exposure factor**	
Autoimmune thyroid disease	242, 245.2
**Exclusion criteria**	
Dry eye disease	370.33, 372.53, 375.15, 710.2
Corneal ulcer	370.0
Recurrent corneal erosion	371.42
Corneal scar	371.0
Interstitial and deep keratitis	370.5
Corneal neovascularization	370.6
Ocular burn	940
Open globe injury	871
**Coexisting disease**	
Hypertension	401-405
Diabetes mellitus	250
Coronary artery disease	410-414
Chronic obstructive pulmonary disease	490, 491, 496
Chronic liver disease	571
Chronic kidney disease	585
Cerebrovascular disease	430-438
Depressive disorder	296.2, 296.3
Anxiety disorder	300
Sleeping disorder	307.41, 307.42, 327.0, 780.50, 780.52, 292.85
Cancer	140-208, 230-234
**Study outcome**	
Dry eye disease	370.33, 372.53, 375.15, 710.2
Sjögren's syndrome	710.2
Corneal ulcer	370.0
Recurrent corneal erosion	371.42
Corneal scar	371.0
