# Supplementary figures and images for: The Association between Autoimmune Thyroid Disease and Ocular Surface Damage: A Retrospective Population-Based Cohort Study
Source: J Clin Med. 2023 Apr 29;12(9):3203. doi: 10.3390/jcm12093203 (PMC10179488; doi:10.3390/jcm12093203)

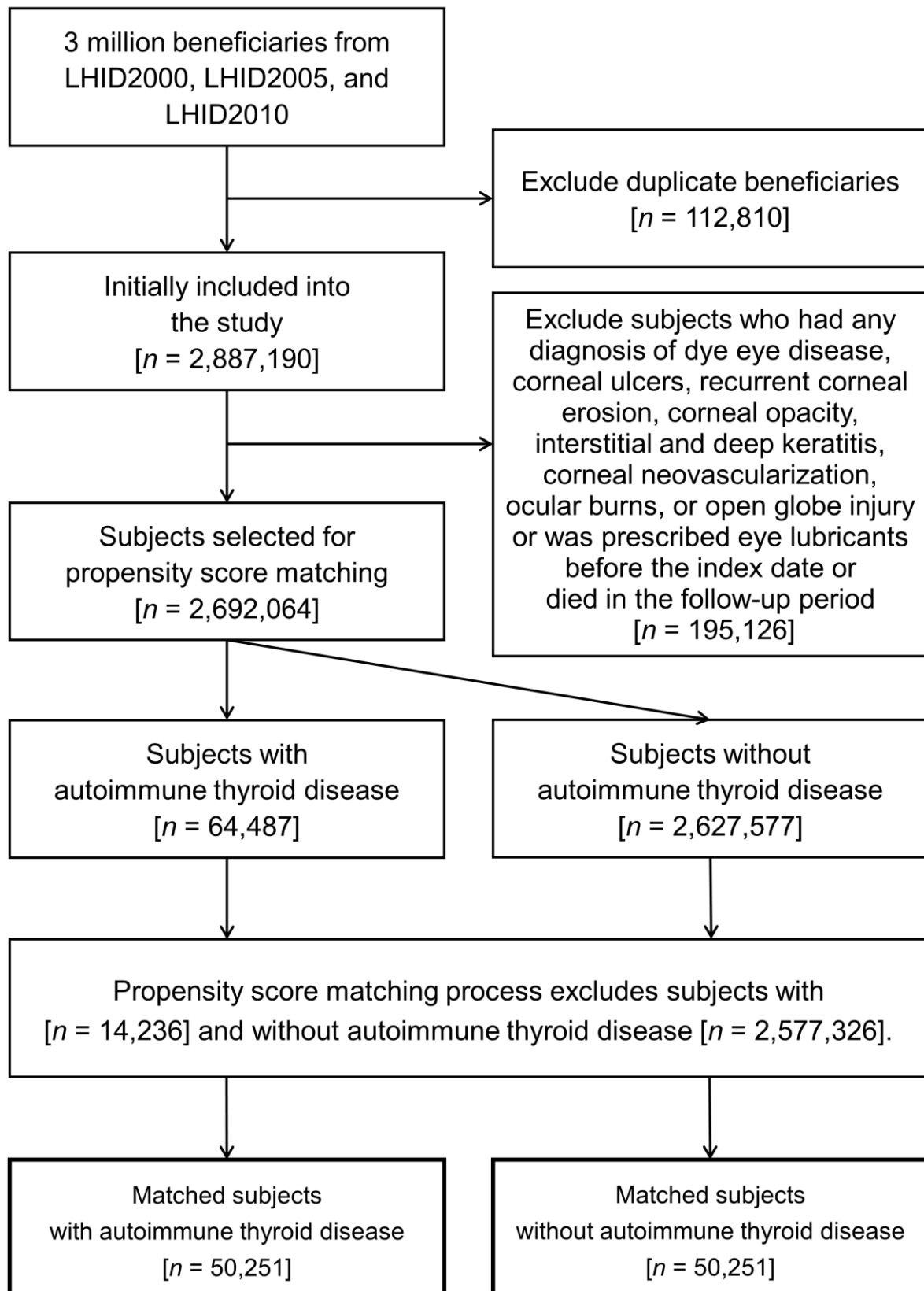

Figure S1. Flow diagram for patient selection.

Supplement: Supplementary file 1 [file jcm-12-03203-s001.zip › Supplementary Figure S1.pdf]
